# O-GlcNAc as an Integrator of Signaling Pathways

**DOI:** 10.3389/fendo.2018.00599

**Published:** 2018-10-16

**Authors:** Qunxiang Ong, Weiping Han, Xiaoyong Yang

**Affiliations:** ^1^Department of Comparative Medicine, Yale University School of Medicine, New Haven, CT, United States; ^2^Program in Integrative Cell Signaling and Neurobiology of Metabolism, Yale University School of Medicine, New Haven, CT, United States; ^3^Department of Cellular and Molecular Physiology, Yale University School of Medicine, New Haven, CT, United States; ^4^Laboratory of Metabolic Medicine, Singapore Bioimaging Consortium, Agency for Science, Technology and Research (A^*^STAR), Singapore, Singapore

**Keywords:** O-GlcNAc transferase, O-GlcNAcase, signaling integrator, homeostasis, O-GlcNAcylation, spatiotemporal dynamics, metabolic sensor, posttranslational modification

## Abstract

O-GlcNAcylation is an important posttranslational modification governed by a single pair of enzymes–O-GlcNAc transferase (OGT) and O-GlcNAcase (OGA). These two enzymes mediate the dynamic cycling of O-GlcNAcylation on a wide variety of cytosolic, nuclear and mitochondrial proteins in a nutrient- and stress-responsive fashion. While cellular functions of O-GlcNAcylation have been emerging, little is known regarding the precise mechanisms how the enzyme pair senses the environmental cues to elicit molecular and physiological changes. In this review, we discuss how the OGT/OGA pair acts as a metabolic sensor that integrates signaling pathways, given their capability of receiving signaling inputs from various partners, targeting multiple substrates with spatiotemporal specificity and translocating to different parts of the cell. We also discuss how the pair maintains homeostatic signaling within the cell and its physiological relevance. A better understanding of the mechanisms of OGT/OGA action would enable us to derive therapeutic benefits of resetting cellular O-GlcNAc levels within an optimal range.

## Introduction

Proteins are extensively post-translationally modified, with over 450 different types of posttranslational modifications (PTM) that play important roles in cellular signaling, protein-protein interactions or modulation of gene expression (1). The availability of such a PTM code, in addition to spatiotemporal dimensions, provides cells with greater dynamic range to respond to a myriad of stimuli and cellular environment (2). O-GlcNAcylation is a prevalent PTM that is found on serine and threonine residues of proteins in the nucleus, cytoplasm and mitochondria (3, 4). Up to 4,000 and potentially more proteins have been found to be O-GlcNAcylated (5). Remarkably, there is only one enzyme, O-GlcNAc transferase (OGT) (6, 7), that catalyzes the addition of O-GlcNAc to proteins and one that removes the modification, namely O-GlcNAcase (OGA) (8).

**Figure 1:**
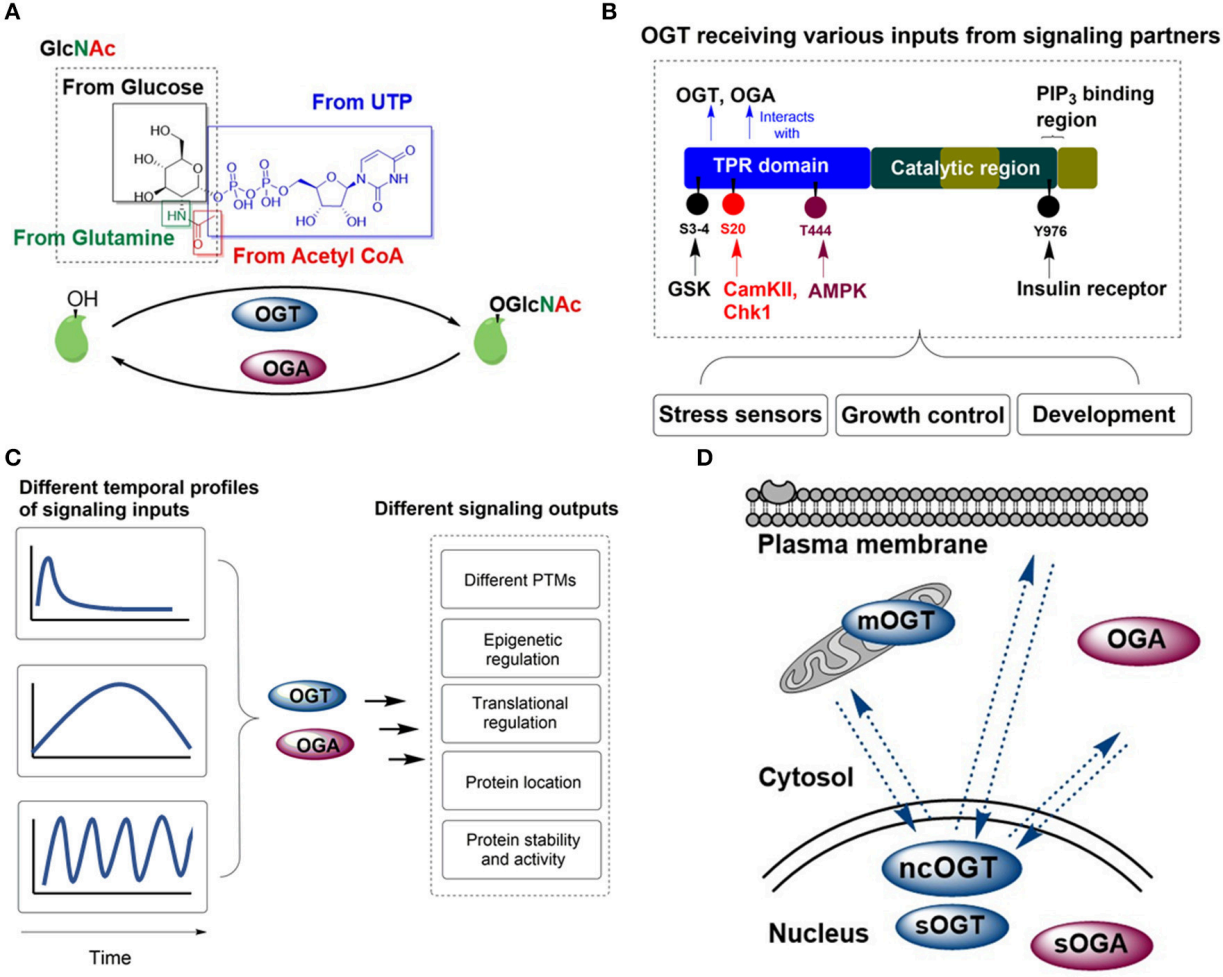
OGT/OGA enzyme pair acts as an integrator for cellular signaling. **(A)** OGT and OGA catalyze the addition and removal of O-GlcNAc on proteins, respectively. The availability of UDP-GlcNAc, the product of amino acid, carbohydrate, fatty acid, nucleotide and energy metabolism, is crucial for OGT function. **(B)** OGT can be phosphorylated by signaling partners (such as AMPK, CamKII, Chk1, GSK, and the insulin receptor), which integrates various inputs and results in O-GlcNAcylation of protein substrates involved in diverse biological responses. **(C)** Temporal control is critical for O-GlcNAcylation of protein subtrates, where OGT/OGA activity is responsive to signaling input changes. **(D)** A unique feature of O-GlcNAcylation is the ability of OGT to shuffle in and out of the nucleus. OGT and OGA isoforms are present in the cytosolic, nuclear and mitochondrial space, whereby the spatial control of signaling can be achieved efficiently.

O-GlcNAcylation uses the substrate UDP-GlcNAc, the final product of nutrient flux through the hexosamine biosynthetic pathway (HBP) which integrates amino acid, carbohydrate, fatty acid, nucleotide, and energy metabolism (Figure 1A). The HBP fluctuates with cellular metabolism and may be dramatically altered under physiological and pathological conditions. The extent of O-GlcNAcylation can reflect metabolic dynamics in the cell. This sets up the OGT/OGA pair to be an “all-in-one” metabolic and nutrient sensor and has been understood to alter diverse cellular processes such as apoptosis, gluconeogenesis, calcium signaling, insulin signaling, and mitochondrial homeostasis. Physiologically, disruption of OGT and OGA function has been implicated in the pathogenesis of several major health problems, such as diabetes, cancer and neurodegenerative diseases (9). Constitutive deletion knockout of the *ogt* and *oga* gene causes early postnatal lethality in mammals (10, 11).

Remarkable progress in understanding the signaling properties of O-GlcNAcylation has suggested that O-GlcNAcylation and the OGT/OGA enzyme pair may play a key regulatory role in coordinating cellular signaling (3). In this Review, we explore major concepts regarding the role of OGT and O-GlcNAcylation and provide a conceptual understanding of how OGT can potentially act as a metabolic sensor to integrate signaling inputs transduced by other signaling components in the cell.

Traditionally, intracellular signaling has been thought of as linear modules of signaling architecture, from activation of receptors via ligands to information flow through phosphorylation cascades and subsequent activation of transcription and protein synthesis. However, recent advances in mass spectrometry and proteomics have allowed a more holistic assessment of protein and lipid modifications that result from singular events, and the results have indicated that a single stimulus could result in complex responses with multiple pathways being activated simultaneously with feedback loops and cross-talks in place (12, 13). The spatiotemporal dynamics of signaling also contributes to the complexity of the system.

## OGT/OGA pair as a signal integrator

A simple way to re-think about signaling proteins and their organization would be to identify key signaling hubs, termed “integrators.” A signaling integrator within the cellular environment could be defined as a protein complex which could receive multiple forms of inputs from other signaling molecules, elicit multiple outputs simultaneously with spatiotemporal control and the ability to reset itself in a timely fashion. Its role would be critical in allowing quick coordination of the activity of several signaling modalities and modulating their signaling dynamics to provide a homeostatic balance or amplification of signaling intensity. While the idea that several signals converge on a specific substrate to elicit defined biological events is a common theme in cellular regulation, the existing ideas prevalent in the field focusses primarily on specific activities of effectors downstream and is limited on the scale of proteome involved. Ideally, the most effective and efficient signal integration would be able to take place almost instantly, allowing the cells to effect quick responses upon gathering external stimuli via global modification and activation of proteins and genetic machinery. The OGT/OGA signaling complex possesses the above-mentioned characteristics that allow them to be an ideal integrator of signaling inputs within cells.

### An all-encompassing metabolic sensor

While the substrate of OGT, UDP-GlcNAc, integrates information about nutrient flux within the cell, OGT and OGA are also uniquely positioned to receive information from several key nutrient-sensitive signaling pathways and appropriately transduce this information.

One of the prevailing views in the field is that OGT makes use of its N-terminal tetratricopeptide repeats (TPR) domain, which is an extended superhelical structure of up to 13.5 TPRs, to act as a scaffold for interacting substrates (14). Besides allowing substantial binding plasticity for its downstream signaling substrates, the TPR domain is subject to different posttranslational modifications that regulate its activity, including phosphorylation from adenosine-monophosphate-activated protein kinase (AMPK), calcium/calmodulin-dependent protein kinase II (CaMKII) and insulin-regulated mitotic protein glycogen synthase kinase (GSK3) (Figure 1B).

AMPK is an energy sensor that maintains cellular energy level with regard to cellular stress and nutrient availability. Upon its activation, AMPK phosphorylates OGT at threonine 444, resulting in the dissociation of OGT from the chromatin and inhibition of gene expression. Conversely, OGT could mediate O-GlcNAcylation of AMPK, and positively regulate AMPK activity through its phosphorylation at threonine 172 (15, 16). Glucagon signaling is also intricately linked with O-GlcNAcylation activity of OGT, where CaMKII directly activates OGT by phosphorylation at serine 20 (17). On the other hand, in hyperglycemia, CaMKII can be O-GlcNAcylated and activated at serine 279 autonomously, resulting in persistent activation of CaMKII even after the level of calcium declines (18). In the case of AMPK/OGT and CaMKII/OGT signaling, this type of cooperative signaling facilitates feedback mechanisms and allow signaling events to propagate. Checkpoint kinase 1 (Chk1) is also found to induce OGT phosphorylation at serine 20, which stabilizes OGT and is required for cytokinesis (19).

OGT is well known to be involved in insulin-mediated signaling pathways. One of the downstream partners, GSK3β, has been shown to phosphorylate OGT at serine 3 or 4, which leads to increased OGT activity and potential reciprocal regulation (20). Other than the N-terminus, the C-terminal domain of OGT also plays a role in receiving information from protein partners. Insulin stimulation enhances tyrosine 976 phosphorylation of OGT by the insulin receptor and promotes OGT activity (21). Looking further downstream of the insulin receptor, OGT is identified to bind to phosphatidylinositol-3,4,5-trisphosphate (PIP_3_) while not possessing the pleckstrin-homology domain like PDK1 and AKT, suggesting different binding affinity toward PIP_3_ production (22). This allows OGT to moderate insulin-mediated signaling transduction within 30 min of activation (22). After insulin signaling activation, OGT will be recruited to the plasma membrane where it O-GlcNAcylates and attenuates the multiple components of the insulin signaling pathway (23).

Comparatively, the functions and implications of OGA posttranslational modifications have been less well-studied (as previously reviewed (24, 25). OGA can be O-GlcNAcylated at serine 405 and act as a substrate of OGT (26); however, the implications of O-GlcNAcylation of OGA are unexplored.

### Modulation of major signaling pathways in the cell

O-GlcNAcylation has been well described in previous reviews to regulate and modulate diverse signaling pathways, thus positioning it as an excellent integrator of signals to many effectors within the cell and regulate a wide variety of cellular processes (3, 27–29). O-GlcNAcylation can trigger changes in protein activity, stability and subcellular localization, thereby facilitating signal transduction and propagation. In addition, the dynamic interplay between O-GlcNAc and other posttranslational modifications gives rise to the enormous diversity in signaling modules, which can be mainly classified as reciprocal same-site occupancy and different-site occupancy (29). For the former, phosphorylation and O-GlcNAcylation occur on the same serine and threonine residues and compete with each other. For the latter, the occupancy of one protein region by O-GlcNAc can influence PTMs in another region, and thus affect protein function. The focus in this section is to review and consolidate several exciting new areas in which O-GlcNAcylation plays a key role (Figure 1B).

#### O-GlcNAcylation of stress sensors

Global O-GlcNAc levels often show drastic changes in response to cellular stress including heat shock, hypoxia and nutrient deprivation. The targets and functional consequences of stress-mediated O-GlcNAcylation are beginning to be unveiled (3).

Sirtuin 1 (SIRT1) has been established to be a stress sensor (30). Upon genotoxic, oxidative or metabolic stress, SIRT1 is able to deacetylate proteins that regulate stress responses, such as p53 (31) and NF-kB (32). O-GlcNAcylation of SIRT1 at Ser 549 directly increases its deacetylase activity *in vitro* and *in vivo* and protects the cells from apoptosis (33). A recent study showed that an overall increase in O-GlcNAc levels in breast cancer cells reduces SIRT1 levels and activity in an AMPK-dependent manner. This leads to a decrease in SIRT1-mediated proteosomal degradation of oncogenic transcription factor FOXM1, which promotes cancer cell metastasis (34).

Upon glucose deprivation, chromatin-associated fumarase (FH) is phosphorylated by AMPK at Ser 75, which triggers its association with ATF2 and facilitates gene expression for cell growth arrest (35). In cancer cells, upregulated OGT activity results in O-GlcNAcylation of FH at Ser 75. This impedes FH binding to ATF2 under glucose deficiency and confers survival advantage to these cancer cells (35).

#### O-GlcNAcylation of Hippo/YAP pathway in growth control

The Hippo/YAP pathway controls organ size in animals and its dysregulation results in cancer (36). Under glucose-rich conditions, O-GlcNAcylation of YAP at Ser 109 by OGT prevents YAP phosphorylation at adjacent Ser 127 and allows YAP translocation into the nucleus. This facilitates expression of genes for proliferation while inhibiting those genes for apoptosis. As a result, glucose-induced YAP O-GlcNAcylation promotes tumorigenesis (37). This study reveals the functional importance of OGT in the Hippo pathway and growth control, further supporting a prevalent role for O-GlcNAcylation in major signaling pathways.

#### O-GlcNAcylation for development and differentiation

O-GlcNAcylation has been known to regulate the activity of proteins involved in embryonic stem cell (ESC) pluripotency and differentiation, such as SOX2 and OCT4 (38). It has also been shown that O-GlcNAcylation is especially important for brain development, where many proteins for neuronal signaling and synaptic plasticity are O-GlcNAcylated (39, 40). OGT inhibition is recently shown to promote human neural cell differentiation (41). In *Oga* knockout models, anatomical defects include delayed brain differentiation and neurogenesis with pronounced changes in expression of pluripotency markers (42). These studies reinforce the importance of OGT and OGA in neural development and function.

### Temporal dynamics of O-GlcNAcylation

For comprehensive integration of signals coming from different signaling partners, timing is an important aspect that is gradually recognized to be extremely important for signal transduction. One important feature of PTMs is that they should enable cells to respond quickly to cues in a reversible fashion, and that these signals could be defined for their intensity and duration (Figure 1C). Protein O-GlcNAcylation can be transient, persistent or periodic. These temporal modules can affect different signaling outputs such as protein location, activity, and genetic/epigenetic regulation.

Some studies have indicated the dynamic changes of O-GlcNAcylation during insulin signaling (22, 23), lymphocyte activation (43), calcium stimulus (44) and neuronal depolarization (45). In these scenarios, the fluctuation of O-GlcNAcylation occurs in the order of a few minutes, which indicates that the OGT/OGA signaling integrator is sensitive to intricate regulation of each other. Such dynamic ability of the OGT/OGA complex to influence the O-GlcNAcylation levels in the cell makes it an ideal integrator.

Other than short-term regulation, O-GlcNAcylation can result in long-term changes within the cell, which has been elaborated in many previous reviews (3, 28, 29, 46). O-GlcNAcylation or de-O-GlcNAcylation of proteins may result in their activity and stability to be shifted. O-GlcNAcylation may also regulate transcription and epigenetic programs, engaging in diverse protein complexes in a context-dependent manner to produce longer-term changes within the cell.

### Subcellular distribution and translocation of OGT/OGA

Signal transduction is profoundly non-uniform in space, and the space in which signaling activities are carried out creates a code for signaling specificity. Alternative splicing results in three variants of OGT, namely nucleocytoplasmic OGT (ncOGT), mitochondrial OGT(mOGT) and short form OGT(sOGT). The longest OGT isoform, ncOGT, is mostly localized in the nucleus but is able to shuttle toward the cytoplasm and plasma membrane in response to signaling cues (7, 47) (Figure 1D). One prime example is the recruitment of OGT from the nucleus toward the plasma membrane upon prolonged insulin activation and PIP_3_ production (22). OGT is also found to alter its nuclear localization upon acute AMPK activation (15). The mechanism underlying how OGT can be localized in both nucleus and cytosol has recently been elucidated where three amino acids (DFP; residues 451-453) in OGT is able to act as a nuclear localization signal. In addition to the DFP sequence, O-GlcNAcylation of the TPR domain of OGT is required for its direct nuclear translocation (48). The ability to translocate between different cellular locations places OGT at a unique position in coordinating signaling activities within different cellular compartments.

Two isoforms of OGA have been identified and characterized. The long isoform of OGA resides mainly within the cytosol (49), whereas the short isoform (sOGA) localizes at the nucleus and lipid droplets (50). *In vitro* experiments indicate that sOGA exhibits lower enzymatic activity compared to OGA (51, 52). A recent structural study has suggested that these two isoforms may be distinguished by the ability of OGA, but not sOGA, to form dimers (53). The varying location and activity of these OGA isoforms add another layer of complexity in regulation of O-GlcNAc signaling (Figure 1D).

## Levels of O-GlcNAcylation signal physiological needs

As in our proposed integrator model, the OGT/OGA complex integrates many signals to effect modifications on proteins, allowing it to achieve metabolic homeostatic control. Some of these modifications may be transient. Yet in many cases, persistent O-GlcNAcylation is required in several proteins, and chronic disruption in O-GlcNAcylation of these proteins may have serious consequences for the cell and the organism. One prime example is the hyperphosphorylation of tau, which is involved in the pathogenesis of Alzheimer's Disease (54).

**Figure 2:**
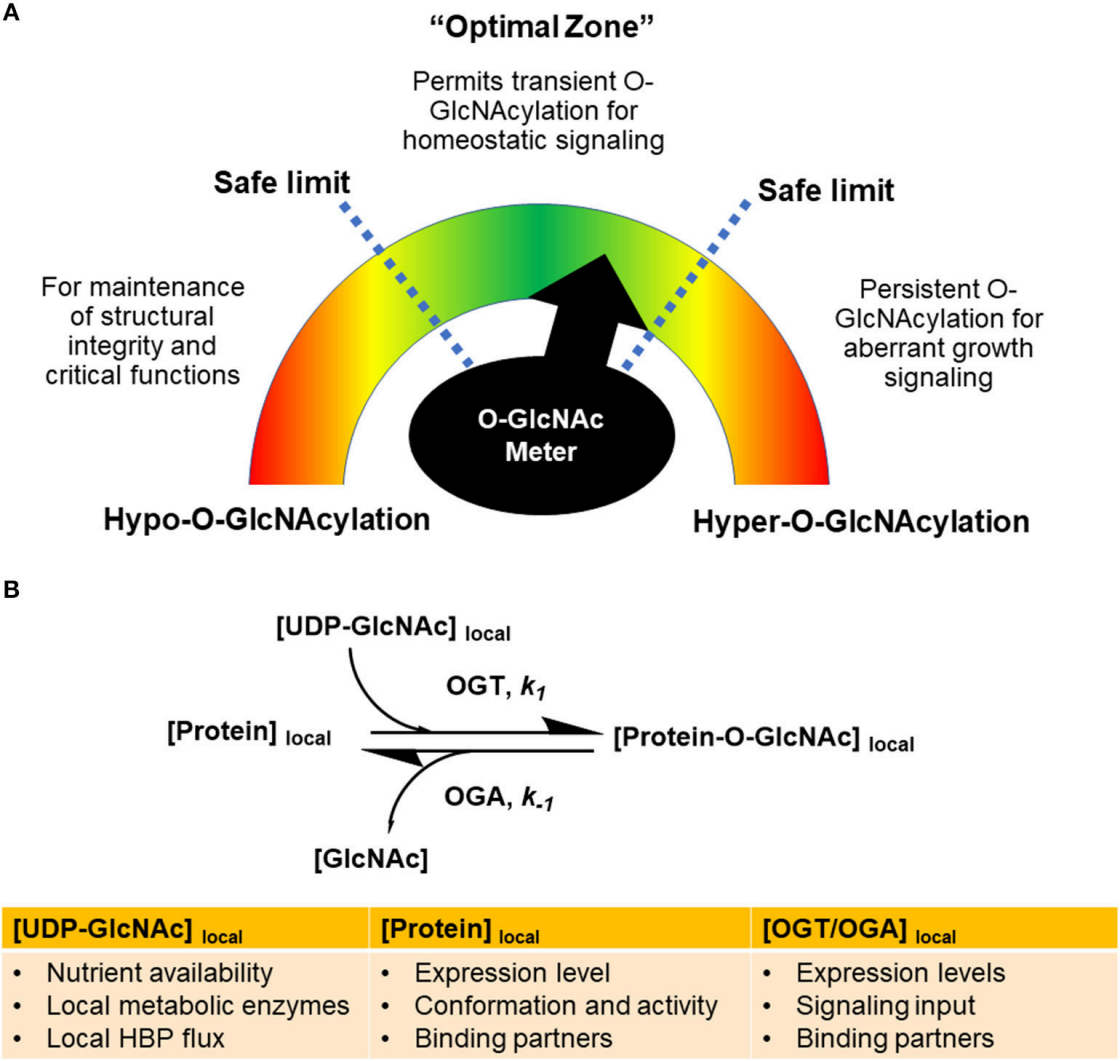
Global and local levels of O-GlcNAcylation gauge signaling responses. **(A)** Global O-GlcNAcylation levels have to be maintained within an optimal range as depicted by an “O-GlcNAc meter” in analogy to a speedometer. Hyper-O-GlcNAcylation, as a result of hyper-OGT activity or high UDP-GlcNAc levels, would result in persistent O-GlcNAcylation of growth signaling proteins, leading to human diseases such as cancer. Hypo-O-GlcNAcylation would impair the structural integrity of protein substrates and their relevant functions. **(B)** The rates of O-GlcNAcylation and de-O-GlcNAcylation of specific proteins, denoted by *k*_1_ and *k*_−1_, are tuned by the local microenvironment. The reactions are determined by local concentrations of UDP-GlcNAc, protein substrates, binding partners, and OGT/OGA activity. Signaling inputs converging on posttranslational modifications of OGT and OGA dictate *k*_1_ and *k*_−1_ of specific proteins.

To reconcile the diverse functions and roles of O-GlcNAcylation, it is important to consider the concept of O-GlcNAc homeostasis occurring only at certain cellular O-GlcNAcylation levels within the safe limits (Figure 2A). In the “optimal zone” of signaling, O-GlcNAcylation levels are well buffered within a certain range of activity (3, 9). A minimum level of O-GlcNAc levels would be present to ensure O-GlcNAcylation of proteins for their proper functions, while there will be a variable component of O-GlcNAc levels tuned by the OGT/OGA pair for signaling. To further enhance the effectiveness of this pool of O-GlcNAc, the cells could control OGT/OGA activity in the spatiotemporal dimensions to amplify or dampen cellular responses.

Hypo-O-GlcNAcylation usually arises from low UDP-GlcNAc levels or a dramatic imbalance between OGT/OGA expression and activity. Given the observation that some of OGT's protein substrates are constitutively modified at physiological UDP-GlcNAc levels while some vary widely (55), it can be envisioned that the cells would preferentially feed O-GlcNAc moieties toward protein residues that are required for essential structural and functional integrity of the protein (56). At this point, dynamic O-GlcNAc signaling may dampen its amplitude and achieve limited effectiveness. In the event of persistent hypo-O-GlcNAcylation, proteins would then be prone to structural changes without the protective effects from O-GlcNAcylation, thus manifesting states that may have deleterious effects for the cell.

On the other hand, in the hyper-O-GlcNAcylation regime, serine or threonine residues of proteins may shift the equilibrium of phosphorylation toward O-GlcNAcylation. In many types of cancer, tumor cells have altered glucose metabolism, resulting in higher hexosamine flux toward UDP-GlcNAc (57, 58). Oncogenes such as Myc (59, 60) and NF-kB (61) are stabilized with persistent O-GlcNAcylation, thus promoting cancer growth. As such, in an extended duration of hyper-O-GlcNAcylation, O-GlcNAc cycling is not well-buffered and homeostatic signaling by OGT/OGA can be compromised.

It is important to note that O-GlcNAcylation and nutrient availability is not always in a linear relationship. Numerous studies have reported a global increase in cellular O-GlcNAcylation in response to nutrient starvation, a condition in which low UDP-GlcNAc levels are expected (35, 62–64). Furthermore, OGT has preferential selectivity for certain substrates under different UDP-GlcNAc concentrations. For instance, O-GlcNAcylation of PGC-1α peaks at 5 mM glucose and is lower under hypo- and hyperglycemic conditions, indicating that other factors are involved in the nutrient sensitivity of this modification (65). Overall, O-GlcNAc homeostasis is determined by nutrient availability, OGT/OGA expression and activity, protein substrate selectivity, as well as other co-factors (Figure 2B).

O-GlcNAcylation of specific proteins can be described as an equilibrium between the forward reaction driven by OGT (denoted as *k*_1_) and the reverse reaction driven by OGA (denoted as *k*_−1_) (Figure 2B). This equilibrium is dictated by the local microenvironment, including local concentrations of UDP-GlcNAc, protein substrates, and OGT/OGA complexes. Local UDP-GlcNAc levels are affected by the availability of extracellular nutrients, the local activities of metabolic enzymes and the HBP flux rate. In addition to their local concentrations, the conformation and activity of protein substrates, OGT and OGA, and binding partners can have a profound influence on *k*_1_ and *k*_−1_. In particular, OGT and OGA can receive various signaling inputs in the form of posttranslational modifications that modulate *k*_1_ and *k*_−1_ of specific proteins (Figures 1B, 2B).

## Machinery maintaining O-GlcNAc homeostasis

Given that O-GlcNAcylation levels of specific proteins may not be linearly correlated with nutrient availability, the expression and activity of OGT and OGA have to be tightly regulated. The observation that the levels of OGT and OGA transcripts and proteins fluctuate in many processes, such as cell cycle progression, stress response and tissue development, hint at the intricacies of this regulatory machinery at work (24, 66).

Studies have demonstrated that the levels of OGT and OGA would compensate for one another. Upon OGT knockout, inhibition or knockdown, OGA protein levels are reduced (67). This might be a consequence of reducing O-GlcNAc levels within cells, and OGA protein levels are sensitive to such changes at the posttranscriptional level. It has also been suggested that the *Oga* gene is situated within the highly conserved NK homeobox gene cluster. Since this region is targeted by the PcG repressor complex which comprises OGT, it is likely that OGT modulates OGA expression at the transcriptional level (68). Conversely, upon OGA inhibition, OGT levels are downregulated while OGA levels are upregulated (69). Our recent work provides mechanistic insight into mutual regulation of OGT and OGA at the transcriptional level. Specifically, we found that OGA acts as a co-activator that directly promotes OGT transcription through cooperation with C/EBPβ and p300 histone acetyltransferase (70). Another recent work has suggested that there is a conserved OGT intronic splicing silencer that is necessary for OGT intron retention (71). The OGT intron retention is dynamically sensitive to cellular O-GlcNAc levels. At high O-GlcNAc levels, the intron is retained and results in the degradation of OGT transcripts. However, upon low O-GlcNAc levels, the intron is spliced out and OGT proteins are produced, thereby increasing its protein level. These studies highlight the multiple layers of regulation of OGT and OGA expression to ensure O-GlcNAc homeostasis.

While the OGT and OGA levels are well controlled by intricate transcriptional and posttranscriptional mechanisms, it is likely that such regulation can only exist within a well-tolerated “optimal zone” for a limited period of time. Chronic hypo- or hyper-O-GlcNAcylation would potentially undermine the effectiveness of O-GlcNAc signaling, thus contributing to the pathogenesis of human disease.

## Perspectives and future directions

While the promiscuity of OGT substrate recognition has made it technically challenging to define the specificity of OGT action, it represents a huge ground of opportunities to be exploited. More tools and technologies have to be developed to understand O-GlcNAc signaling. Due to the diversity of substrates that OGT and OGA can act on to regulate cellular function, and the complex compensatory pathways that could take place, delineating the cause and effect remains a major challenge. Future studies are required to investigate the “O-GlcNAc proteomics” with temporal precision and identify potentially the subsets of proteins that are sensitive to different UDP-GlcNAc levels and in different temporal contexts.

One understudied component of O-GlcNAc signaling is the spatial contexts of O-GlcNAcylation. In various cellular compartments of the cell, such as the nucleus, mitochondria, cytosol and plasma membrane, OGT and OGA have different physiological roles. Within each cellular region, there are different potential binding partners for both OGT and OGA. Local concentrations of UDP-GlcNAc may also contribute to O-GlcNAcylation of specific substrates (Figure 2B). Profiling the variability of local UDP-GlcNAc levels and O-GlcNAcylation of proteins in real-time and within individual cells would provide a powerful tool to understand the dynamics of this modification. Developing the tools to visualize the kinetics of OGT/OGA interactions with their signaling partners would also help our understanding of O-GlcNAc regulation in spatial dimensions.

A better understanding of the roles of O-GlcNAylation in diverse signaling pathways would be another major direction to be pursued. Development of site-specific O-GlcNAc antibodies would be essential in accelerating our understanding of the function of specific protein O-GlcNAcylation. As O-GlcNAcylation is tightly linked with different PTMs in the cellular metabolic network, it would be useful to identify the intricate relationships among various types of PTMs on proteins. Crosstalk between phosphorylation and O-GlcNAcylation has been relatively well studied, where O-GlcNAcylation can occur reciprocally or sequentially with phosphorylation on the same or different residues. Such studies could be extended to explore the link between O-GlcNAcylation and other PTMs, such as acetylation, methylation, and succinylation and ubiquitination.

## Conclusions

The cellular signaling machinery is a complex network of components which is only partially understood. The complexity is not only due to the sheer quantity of participants and its high degree of connectivity, but also to the spatiotemporal dimensions of signaling which confers different functions on the same proteins under various contexts. In this review, we have proposed the OGT/OGA pair as a metabolic sensor and an integrator of cellular signaling processes. This relies on the ability of the OGT/OGA complex to receive metabolic and stress signals from multiple upstream partners, and to drive O-GlcNAc modification on diverse sets of downstream targets with precise spatiotemporal control. The OGT/OGA pair is tightly regulated by multiple layers of transcriptional, posttranscriptional and posttranslational control to maintain cellular O-GlcNAc levels within an optimal zone. This “O-GlcNAc meter” ensures O-GlcNAcylation as an effective toolbox to tune and integrate signaling pathways.

## Author contributions

All authors listed have made a substantial, direct and intellectual contribution to the work, and approved it for publication.

### Conflict of interest statement

The authors declare that the research was conducted in the absence of any commercial or financial relationships that could be construed as a potential conflict of interest. The reviewer RT and handling editor declared their shared affiliation at the time of review.
